# Recurrent Network Models for Perfect Temporal Integration of Fluctuating Correlated Inputs

**DOI:** 10.1371/journal.pcbi.1000404

**Published:** 2009-06-05

**Authors:** Hiroshi Okamoto, Tomoki Fukai

**Affiliations:** 1 Laboratory for Neural Circuit Theory, RIKEN Brain Science Institute, Wako, Saitama, Japan; 2 Corporate Research & Technology Development Group, Fuji Xerox, Ashigarakami-gun, Kanagawa, Japan; 3 Department of Complexity Science and Engineering, University of Tokyo, Kashiwa, Chiba, Japan; University College London, United Kingdom

## Abstract

Temporal integration of input is essential to the accumulation of information in various cognitive and behavioral processes, and gradually increasing neuronal activity, typically occurring within a range of seconds, is considered to reflect such computation by the brain. Some psychological evidence suggests that temporal integration by the brain is nearly perfect, that is, the integration is non-leaky, and the output of a neural integrator is accurately proportional to the strength of input. Neural mechanisms of perfect temporal integration, however, remain largely unknown. Here, we propose a recurrent network model of cortical neurons that perfectly integrates partially correlated, irregular input spike trains. We demonstrate that the rate of this temporal integration changes proportionately to the probability of spike coincidences in synaptic inputs. We analytically prove that this highly accurate integration of synaptic inputs emerges from integration of the variance of the fluctuating synaptic inputs, when their mean component is kept constant. Highly irregular neuronal firing and spike coincidences are the major features of cortical activity, but they have been separately addressed so far. Our results suggest that the efficient protocol of information integration by cortical networks essentially requires both features and hence is heterotic.

## Author Summary

Spikes are the words that neurons use for communicating with one another through their networks. While individual cortical neurons generate highly irregular spike trains, coincidently arriving spikes are considered to exert a strong impact on postsynaptic-cell firing and hence to play an active role in neural information processing. However, little is known about whether computations by the brain benefit from such coincident spikes. Here, we show in a recurrent network model that coincident spikes embedded in random spike trains provide a neural code useful for highly accurate temporal integration of external input. In fact, the proposed neural integration is almost perfectly accurate in the mathematical sense. A wide range of cognitive behavior relies on temporal integration. For instance, it is a central player in sensory discrimination tasks and interval timing perception. Our model provides the neural basis for quantitative understanding of animal's decision behavior. In addition, it may account for why cortical activity shows a heterotic feature with irregular firing and synchronous spikes.

## Introduction

The integration of information over time underlies a variety of cognitive and behavioural functions, such as decision making, prediction of upcoming events, or interval timing. For instance, psychology models of decision making hypothesize that temporal integration of a sensory input or an internal signal represents the subjective belief or the likelihood signal for a particular decision [1],[2]. The subsequent action is executed after this signal reaches a certain criterion. Some task-related neuronal activities show gradually increasing firing rates [3]–[8], suggesting that these activities engage in temporal integration [9]–[13].

Results of psychological experiments suggest that the above input is integrated with an equal weight at any time point. For example, animal's decision behavior does not depend on the temporal order of presenting the same set of stimuli, each of which represents a different piece of evidence for decision [14],[15]. This uniformity of temporal integration naturally appears if temporal integration of a constant stimulus has the following properties: (i) the likelihood signal grows linearly with time and (ii) the rate of the linear growth is proportional to the stimulus intensity (i.e., temporal integration by neurons is a linear operation). The temporal integration that fulfills these two properties is termed “non-leaky” or “perfect” temporal integration, which well explains some quantitative aspects of behavior, such as the statistics of saccadic eye-movement and visual short-term memory [14]–[17]. The two properties are obviously satisfied if the likelihood signal 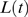 expresses 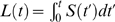 in the mathematical sense, with 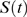 being the stimulus intensity at time 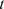 (in particular, 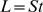 if 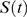 is constant). Although climbing activity has been modeled with network [18]–[23] or single-cell mechanisms [24]–[27], these models did not seriously address the two properties of perfect temporal integration. In fact, it is not trivially easy to construct neural integrator models that satisfy the two properties.

Here, we present a recurrent network model of spiking neurons that performs perfect temporal integration of excitatory and inhibitory synaptic inputs. We show that the two properties (i) and (ii) can be obtained if irregular input spike trains are partially correlated and the correlation component of synaptic inputs represents the quantity integrated by the network. We then analytically prove the perfect integration property of the network model, noting that the partially correlated input spikes modulate the variance, but not the mean, of the synaptic current. The present model is an extensively improved version of our previous model that accounts for the bistable property of climbing and descending activities of anterior cingulate neurons [28]. The previous model, and many other models, integrated the amplitude of a constant or slowly changing external current, and the fluctuations around the mean were merely noise or played only a secondary role [29],[30]. In contrast, the present model suggests that information may be better represented by the fluctuations induced by spike correlations in synaptic input in some neural computations.

## Results

We consider a recurrent network of 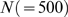 excitatory, leaky integrate-and-fire neurons. The membrane potential of neuron 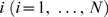 follows the equation:
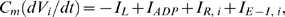
(1)where 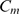 is the membrane capacitance and 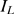 is the leak current. The model neuron displays a long-lasting depolarization current 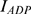 after it discharges a spike [28]. The depolarization leads to further spike discharges, which in turn refresh this current. Consequently, a neuron, once it fires, may remain in the regenerative firing state [31],[32]. In vitro cortical pyramidal cells display spike-triggered, prolonged depolarization induced by the activation of Ca^2+^-dependent cation currents [33]–[35]. In addition, climbing/descending activity displayed bimodal distributions of firing rate in the monkey anterior cingulate cortex [28], suggesting that the response of cingulate neurons may be bistable. Even if single cortical neurons are not bistable, a local neuronal circuit can show a bistable response [19]. Although the bistability of cortical neurons in vivo remains to be clarified, here we employed bistable single neurons for the simplicity of modeling. The excitatory leaky integrate-and-fire neurons are randomly connected by recurrent synapses with a uniform weight, as represented by 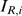 in Equation 1. In addition to the recurrent inputs, each neuron receives partially correlated excitatory and inhibitory synaptic inputs 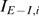 from outside of the network. We did not include inhibitory neurons for the simplicity of mathematical analyses. In numerical simulations, the inclusion of inhibitory neurons reduced the slope of climbing activity without changing the essential property of temporal integration. The details of our model are described in Methods.

All the neurons were initially set in the resting state in each trial of temporal integration. If the external synaptic inputs do not fluctuate, the membrane potentials are frozen at a subthreshold value and the neurons do not fire. In the presence of fluctuations, the membrane potential of each neuron randomly shifts around this value and may eventually reach the threshold to generate a spike, which in turn sets the neuron in the active state. In reality, a single spike may not be sufficient to set a cortical neuron in the active state. For the time being, however, we will employ the oversimplified model to focus on the essential mechanism of temporal integration. Simulations with a more realistic neuron model will be shown later. As time advances, the individual neurons undergo asynchronous transitions from the resting to the active state. Below, we investigate how the number of active-state neurons grows in time.

### Perfect temporal integration of partially correlated synaptic inputs

**Figure 1:**
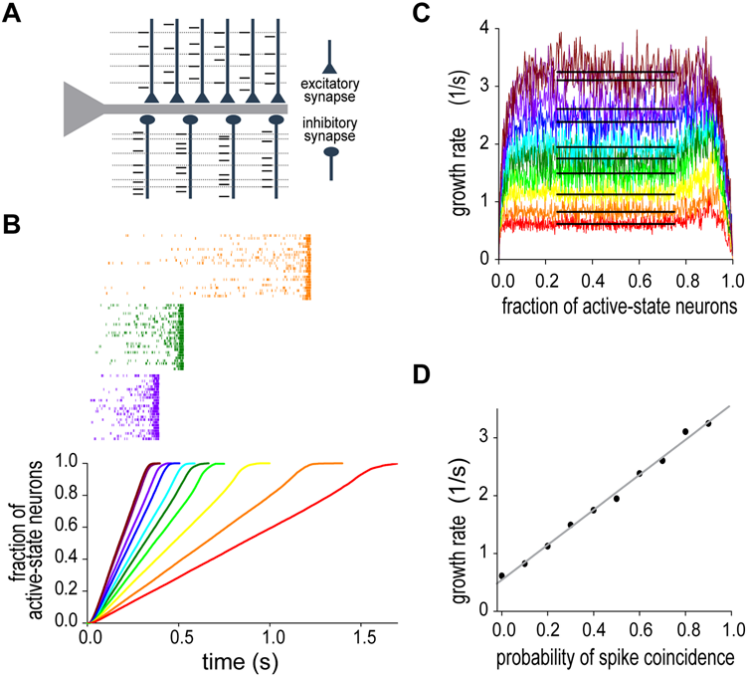
The behavior of the network model integrating the partially correlated synaptic inputs. Our network model represents the moment-to-moment result of perfect temporal integration with the instantaneous number of active-state neurons. (A) Schematic drawing of the partially synchronized synaptic inputs. Both excitatory and inhibitory synapses receive partially coincident spikes (dashed lines). (B) Linear growth of the number of active-state neurons is shown with some examples of spike raster for various values of the probability of spike coincidence. Different colors represent different values of the coincidence probability. The network model was examined by Monte Carlo simulations with explicit treatment of the synaptic gate equation. (C) The growth rate was plotted as a function of the fraction of active-state neurons in the network. In accordance with the linear growth of neuronal activation, the rate stays around a constant value (horizontal black lines) for wide range of the fraction. (D) The rate is a linear function of the probability of spike coincidence. The grey straight line is obtained by the least square method.

We numerically investigated how the number of active-state neurons grows in time and how the slope of climbing activity is modulated by the correlated spikes introduced into 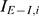. Consider the situation that 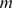 input spikes are coincident with probability 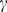 at arbitrary excitatory and inhibitory synapses (Figure 1A); namely, among the 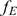 input spikes arriving at any excitatory synapse in 1 second, an average of 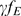 spikes are coincident with spikes at some other 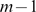 excitatory synapses. A similar condition is fulfilled by the inhibitory synaptic inputs. The combination of 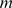 excitatory or inhibitory synapses can vary from coincident event to event. We note that changing the probability of spike coincidences 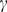 does not change the average rate of input spikes. Correlated spikes are indeed ubiquitous in the brain [36]–[41].

The fraction 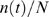 increased at an almost constant rate (Figure 1B and 1C), where 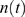 denotes the instantaneous number of neurons in the active state in the neuronal population. More interestingly, the rate of this linear growth was approximately proportional to 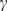 (Figure 1D). Here, the growth rate was defined as 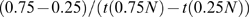 in terms of the time 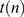 of transition 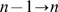 in the network. These results imply that the number of active-state neurons represents the moment-to-moment result of temporal integration of correlated spike inputs, where the linear relationship between 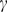 and the slope of activity increase is a signature of perfect temporal integration. We confirmed that essentially the same results can be obtained in a similar recurrent network model with realistic calcium dynamics (Text S1). This model could also generate spontaneous activity in the resting state of neurons.

### Gaussian white-noise approximation of external input

To see the underlying mechanism of perfect temporal integration, we introduce an approximate treatment of partially correlated, yet irregular synaptic inputs. In general, the fluctuation component of excitatory or inhibitory synaptic conductance comprises temporally correlated noise (i.e., colored noise). Here, however, we treat the fluctuation component as Gaussian white noise 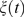 with mean of 0 and variance of 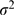, and describe the total synaptic current as
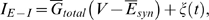
(2)with average total conductance 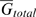 and effective reversal potential 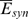 (Material and Methods). Let 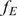 and 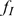 be the rate of presynaptic spikes at an excitatory or an inhibitory synapse, respectively. Then, it is immediately understood that changing the value of 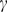 in partially correlated synaptic inputs varies 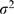, but does not vary 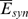 and 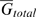, since 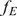 and 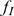 are kept unchanged. We can actually show that 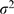 is a linear function of 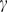 (Equation 9), whereas 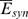 and 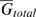 are independent of 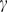 (Equation 8).

**Figure 2:**
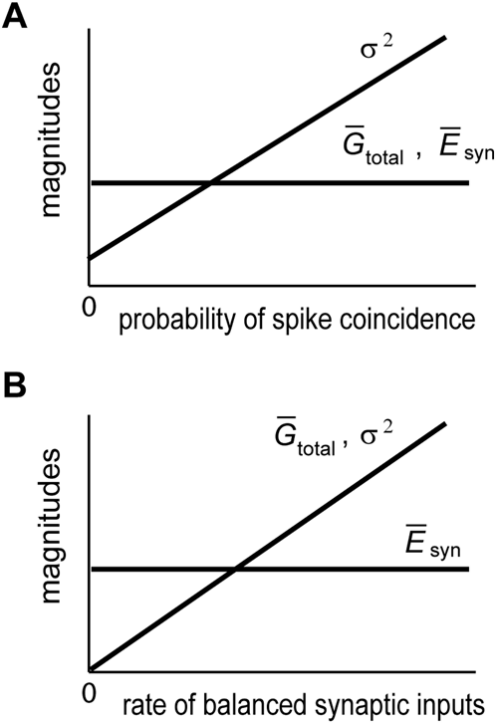
Relationship between partially correlated synaptic inputs and stochastic process. (A) Changes in the average effective reversal potential, average total conductance and the variance of the external inputs are shown schematically as functions of the probability of spike coincidence in partially synchronized synaptic inputs. (B) Similar changes in the same quantities are shown as functions of the rate of balanced excitatory and inhibitory synaptic inputs, when the presynaptic spikes are mutually uncorrelated.

It is noted that such a separate variation of 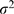 is not obtained by a balanced rate change of excitatory and inhibitory synaptic inputs, which was recently suggested in cortical networks [42],[43]. The balanced rate change, i.e., co-varying 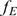 and 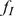 while keeping the ratio 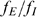 constant, does not change 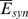, but does change 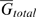 as well as 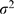 (see Equations 8 and 10). The different effects of partially correlated synaptic inputs and balanced synaptic inputs are schematically illustrated in Figure 2A and 2B, respectively.

The Gaussian white-noise approximation of external input allows us to analytically calculate the average rate of transition, 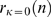, from the resting to the active-state in each neuron (Equation 12 in Material and Methods). By using this rate, we can derive a recursive equation for the rate 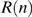 of the state transition 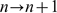,
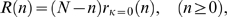
(3)and the mean time 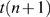 at which the above transition occurs,
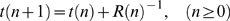
(4)where 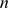 is the instantaneous number of neurons in the active state. Solving Equation 4 with boundary condition 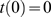, we can obtain the time evolution of 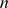.

**Figure 3:**
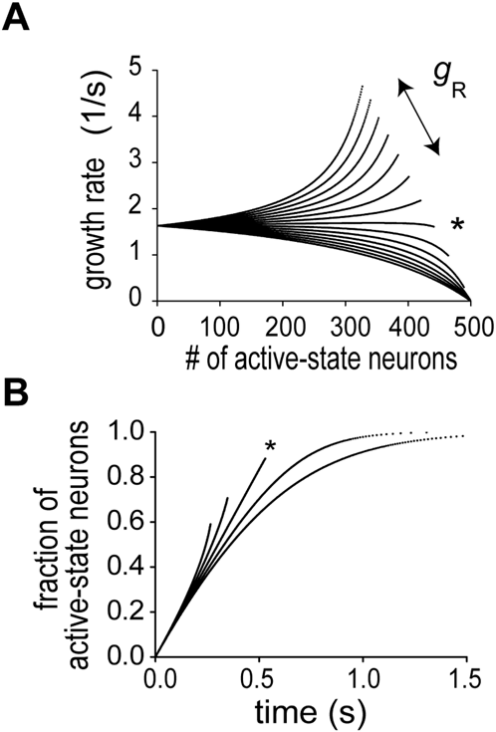
Effect of recurrent excitation on temporal integration of the network model. (A) The growth rate of the number of neurons in the active states is plotted for various values of the maximum conductance of recurrent synapses. The upper the curve is, the larger the conductance is. At an adequate value of the maximum conductance (marked by asterisk), the rate is remarkably constant for a wide range of the number of active-state neurons. (B) Time evolution of active-state neurons is shown for three cases where the maximum synaptic conductance is greater than (upper two), equal to (asterisk) or smaller than (lower two) the adequate value.

In Figure 3A, we calculated the growth rate 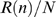 for various values of 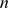 by using Equation 3. For constant growth of 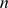, the growth rate should remain constant for an arbitrary value of 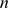. For given values of 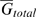 and 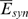, this actually occurs if the strength of recurrent connections, 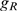, takes an adequate value 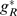 (Figure 3A, asterisk). If 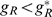, the growth rate decreases monotonically with 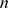, or if 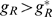, it increases with 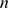 in a highly nonlinear fashion. Accordingly, the time evolution of 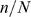 showed clearly different profiles in the above three cases of 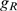 (Figure 3B). It is noted that if the neurons are mutually disconnected, the state transitions in the individual neurons are mutually independent and the growth of 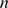 is exponentially decelerated. At 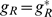, recurrent excitation induced an accelerating effect that counter balances the deceleration effect [28]. All known models of neural integrator require fine tuning of parameter values (in the present case, 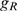) to a certain degree. We argued how this fine tuning may be obtained by our model in a typical behavioral task to generate the presented time interval (Text S1).

### Perfect temporal integration with respect to the variance

**Figure 4:**
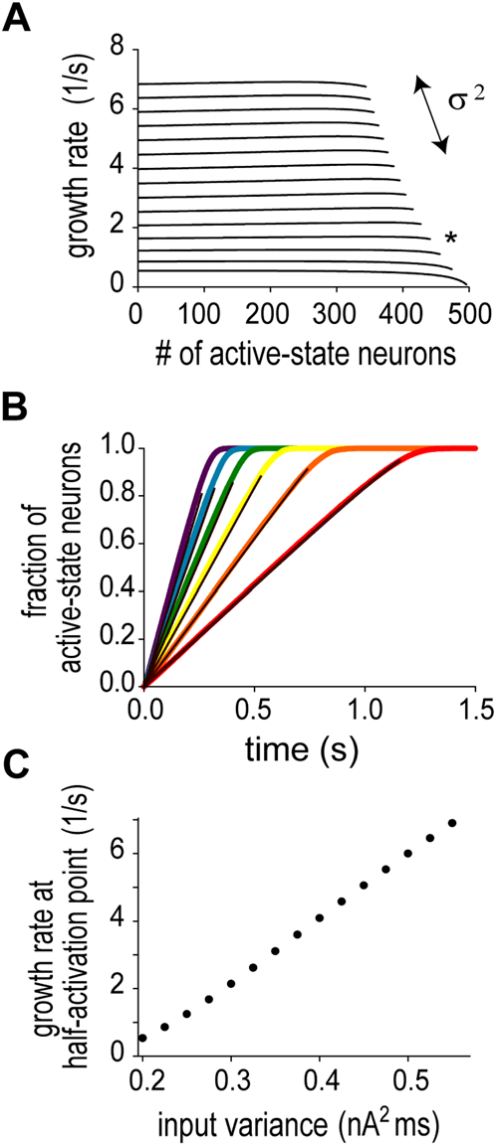
Perfect temporal integration tested by Gaussian white-noise approximation. (A) The constancy of the growth rate found in Figure 3A (asterisk) is held if the variance of the fluctuation component of external current is varied. The greater the variance is, the larger the growth rate is. (B) In accordance with constant rates, the number of active-state neurons grows linearly with time. Black lines indicate analytical results obtained by solving Equation 4, while colored lines indicate the numerical results obtained by Monte-Carlo simulations of Equations 1 and 2. The numerical results were averaged across 100 trials. (C) The dependence of the growth rate on the input variance is linear. The rate was estimated at the time point when the half of neurons was activated.

As shown above, the slope of climbing activity generated by the present network model is proportional to the probability of spike coincidences in partially correlated synaptic input. The Gaussian-white-noise approximation achieves a further insight into the mechanism underlying the perfect temporal integration property. Noting that 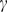 in partially correlated synaptic input is proportional to 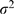, we solved Equations 3 and 4 while varying the value of 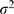, with the values of 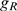, 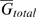 and 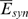 kept unchanged. Surprisingly, the growth rate remains constant in a wide range of 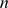, with the constant value depending monotonically on 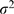 (Figure 4A). In accordance with this, 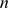 grows linearly with time at the rate determined by 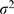 (Figure 4B, black lines). A close inspection of the slope of this linear growth revealed that the growth rate is proportional to 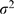 with remarkable accuracy (Figure 4C). These results clearly demonstrate that the instantaneous number of active-state neurons represents the moment-to-moment state of perfect temporal integration of the variance of external stochastic synaptic inputs: 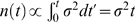 (a more rigorous expression is discussed later). We confirmed that the analytical results qualitatively and quantitatively agree with the numerical results obtained by Monte-Carlo simulations of Equation 1 (Figure 4B, colored lines).

**Figure 5:**
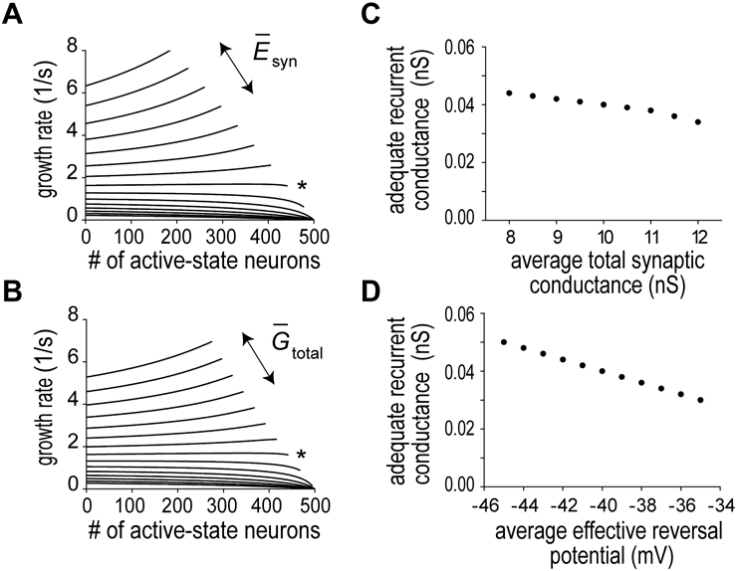
The dependence of the growth rate on the mean part of synaptic current. (A) The rate versus the number of active-state neurons for various values of the effective reversal potential and a fixed value of the total synaptic conductance. The upper the curve is, the larger the reversal potential is. (B) A similar plot for various values of the total synaptic conductance and a fixed value of the effective reversal potential. The upper the curve is, the larger the conductance is. If the parameters determining the mean part are changed, the growth rate is no longer constant for the strength of recurrent connections determined in Figure 3A. (C, D) Values of the maximum conductance of recurrent synapses that yields constant growth rates are shown for different values of the average total conductance or the average effective reversal potential, respectively.

While the above results were obtained for fixed values of 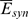 and 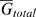, in reality, these quantities may be rapidly modulated if the excitatory-inhibitory balance is changed in external synaptic inputs. In the present model, however, 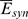 and 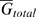 should remain constant during temporal integration. Actually, the growth rate calculated at 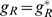 significantly varied with 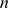, if we varied 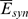 (Figure 5A) or 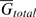 (Figure 5B) while keeping 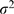 unchanged. Moreover, the value of 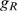 that achieves constant growth rate changes with the value of 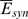 (Figure 5C) or 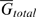 (Figure 5D). Therefore, the present model with fixed 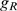 can produce the constant-rate activation of neural population, only if 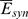 and 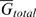 are clamped at constant values during temporal integration. Partially correlated synaptic inputs provide a biologically plausible representation of the fluctuating synaptic inputs that fulfill these conditions.

### Non-leaky property of temporal integration

**Figure 6:**
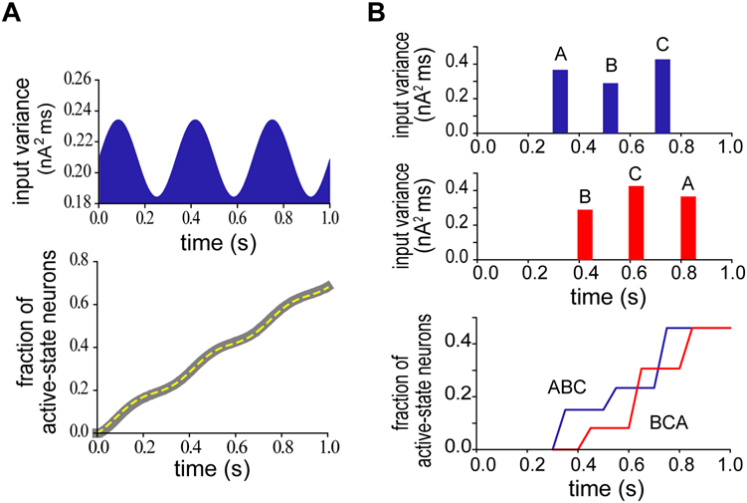
Non-leaky property of the present temporal integration. (A) The integrator network was stimulated by an external fluctuating input with sinusoidal modulations of the variance (*upper*). The network activity (*lower*, *dashed curve*) faithfully integrates a linear function of the variance (*grey curve*), as given explicitly in the text. (B) Three external inputs A, B and C were applied to the integrator network in different temporal patterns (*upper traces*). While the network responded to these sets of stimuli differently, it finally reached the same activation level (*lower*).

The constancy of the growth rate shown in Figure 4A enables our network model to integrate an external fluctuating input with a high precision. In Figure 6A, the variance of the external input was modulated by a sinusoidal function of time during temporal integration. The fraction of active-state neurons in this network represented the integral of a linear function of the variance as, 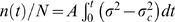, where 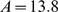 and 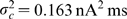. This result demonstrates that the network can perfectly integrate inputs if 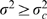 (otherwise, the integration is not really accurate). Consequently, the network model integrates multiple external inputs presented at different time points with an equal weight, irrespective of the temporal order of the presentation. To show this, we applied an identical set of external inputs to the same network as used in Figure 6A in two different temporal patterns (Figure 6B). The network integrated the two patterns of stimuli in different manners, but the final activation of the network was the same since it integrated the same total amount of input. Thus, our model with the properties (i) and (ii) can integrate external inputs in a non-leaky fashion. Note, however, that the microscopic final states generally differ, that is, different combinations of neurons are activated in the final state, since the present temporal integration is essentially stochastic.

### Information decoding in firing rate

**Figure 7:**
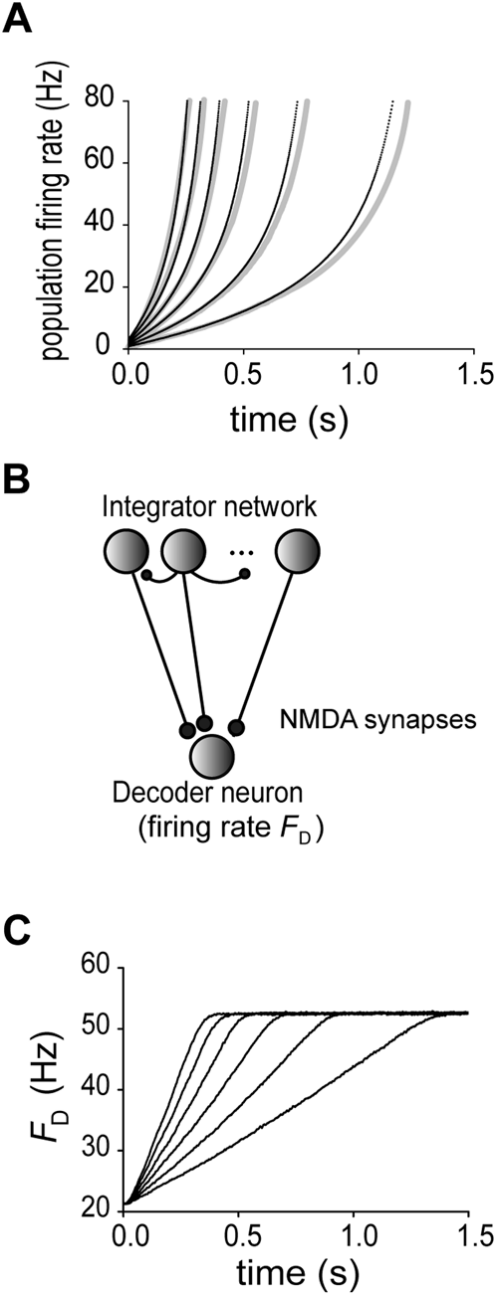
Decoding the output of the temporal integrator network with firing rate. (A) The population firing rate evolves with time in a highly nonlinear fashion. Black and grey lines indicate analytical and numerical results, respectively. (B) Neurons in the integrator network project to a decoder neuron via NMDA excitatory synapses. (C) The firing rate of the decoder neuron evolved constantly with time.

Can downstream neurons read out the network's output represented by the number of active neurons? The number of activate-state neurons would be proportional to the average firing rate of the population of excitatory neurons, if each active-state neuron fired at a constant rate. The firing rate, however, is modulated by growing recurrent synaptic inputs. In fact, the population firing rate displayed a highly nonlinear time evolution (Figure 7A), where the rate was defined as 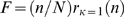 in the analytical treatment and 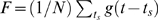 in the numerical simulations. The causal filter 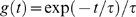 for 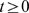 and 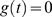 for 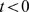 (*τ* = 20 ms), and 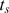 stands for the times at which any neuron fires in the network.

Below, we argue a possible mechanism to decode the number information in firing rate. The decoder neuron is projected to by sufficiently many neurons in the integrator network, and the projections are mediated by NMDA synapses (Figure 7B). If the time course of the NMDA current is sufficiently slow, the amplitude of excitatory postsynaptic potential (EPSP) induced by each active-state neuron eventually saturates and becomes less sensitive to the presynaptic firing rate. Then, the sum of EPSPs may be proportional to the instantaneous number of active presynaptic neurons, so is the firing rate of the decoder neuron (Figure 7C). For successful decoding, we used a relatively slow NMDA current with the decay constant of 200 ms. Such a slow NMDA current was recently reported in experiments [44],[45].

Thus, the nonlinear rate change of integrator neurons is not necessarily an obstacle to decoding the result of temporal integration by downstream neurons. Interestingly, similar nonlinear growth of cortical ensemble activity was recently observed in a multi-unit recording study of temporal interval representation [8], and is consistent with the prediction of our model. We also tested whether the decoding is possible with short-term synaptic depression [46],[47]. Depressing synapses do not transmit information on stationary presynaptic firing rates, so the sum of EPSPs and the firing rate of decoder-neuron might be proportional to the number of active presynaptic neurons. In our simulations, however, this was not the case for experimentally observed ranges of the physical parameters of synaptic depression (results not shown). Depressing synapses did not reach a stationary state rapidly enough.

## Discussion

Neuronal activity recorded from cortical [3]–[8] or sub-cortical [48],[49] regions of behaving animals often exhibits a gradual increase in the firing rate. This so-called climbing activity is considered to reflect the temporal integration of internally or externally driven information necessary for organizing behavior. Previously, we proposed a recurrent network model of stochastic bistable neurons in which a constant external current is integrated with time [28]. In this model, noisy background inputs induce asynchronous transitions of individual neurons between the resting and active states, and the amplitude of the constant external current modifies the transition rate, or equivalently, the slope of the climbing activity. The model was suggested from the bistable neuronal responses observed in climbing activity of anterior cingulate neurons [28]. In the present study, we significantly extended the previous model to improve the accuracy of temporal integration. The novel feature of the present model is that it perfectly integrates the correlation component of external synaptic inputs, when the mean rate of inputs is fixed. We demonstrated that the number of neurons in the active state increases linearly with time and that the rate of this growth is proportional to the probability of receiving coincident presynaptic spikes. It is in this sense that the present temporal integration is perfect.

To obtain perfect temporal integration, the correlations between input spike trains have to be modulated without changing the rates of excitatory and inhibitory synaptic inputs (Figure 1A). The Gaussian-white-noise approximation showed that such an input protocol modulates the variance of synaptic inputs across trials without changing the values of the reversal potential and the total conductance of the synaptic current (Figure 2A). This type of neural code has been suggested in the sound representations of the primary auditory cortex [50]. While synchronization is considered to be important for information coding in the brain [36],[51], how synchronization is utilized by cortical neurons, which typically exhibit irregular neuronal firing, has not been fully clarified. Our results suggest that the instantaneous correlations among irregular synaptic inputs may provide a “fluctuation code” to represent the quantitative information integrated by recurrent networks. We can construct a network model to translate the input firing rate into correlations between the output spike trains. The model will be reported elsewhere. A limitation of the fluctuation code is that fluctuations cannot represent negative input (or negative evidence in decision making) to the integrator network. This limitation, however, is not so serious since positive evidence and negative evidence can be integrated by separate neural networks, which may compete with one another.

It was recently suggested that excitatory and inhibitory synaptic inputs are balanced in cortical neurons [42],[43],[52] and the balanced input has advantage in information representation with irregular neuronal firing [53]. When the net conductance of excitatory synapses is increased on a neuron, the conductance of inhibitory synapses is also increased on the same cell so that their ratio may be kept unchanged. Such balanced rate changes maintain the effective reversal potential of synapses at a nearly constant level, but they change the total synaptic conductance (Figure 2B). For example, if the inputs are given as mutually independent Poisson spike trains, doubling 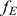 and 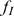 approximately doubles the variance in Equation 10 while keeping the reversal potential unchanged. This manipulation, however, also doubles the total conductance in Equation 8a, which would significantly affect the linear growth of the number of active-state neurons (Figure 5B). Therefore, synaptic inputs with balanced rate changes in excitation and inhibition (and without correlations) do not provide the fluctuation code used in the present model.

Our model represents the moment-to-moment result of perfect temporal integration with the instantaneous number of active-state neurons, 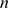. Since a simple integration process may be effectively achieved by single neurons [26],[35], using a population of neurons for such computation seems to be non-economic. The population-based computation, however, makes temporal integration robust against noise or impair of single cells. Temporal integration in our model is particularly robust against such obstacles because the integration is essentially a random process without relying on a precise temporal order of cell activation.

To our best knowledge, climbing activity appears when the activity recorded from a single neuron is averaged over trials. Our model exhibits climbing activity both when activity of a single neuron is averaged over many trials and when activity in a single trail is averaged over many neurons. Therefore, our model supports the hypothesis that population climbing activity plays a crucial role for decision making or temporal integration in single trials. This hypothesis, however, should be examined by simultaneous recording or imaging of climbing activity of massively many neurons in behaving animals.

Contrary to the linear growth of 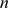, the population firing rate evolves in a highly nonlinear fashion (Figure 7A). Interestingly, such accelerating growth of cortical ensemble activity was recently observed in a multi-unit recording study of the cortical representation of temporal interval [8]. Since neurons communicate with each other through spike exchange, these findings question whether perfect integration can be read out from the population firing rate. In the present study, we demonstrated a possible neural mechanism of the information decoding, by using NMDA receptor-mediated synaptic current with a decay constant (∼200 ms) slightly longer than usual (Figure 7B). The decay constant of NMDA synapses was shown to be two-fold larger in the prefrontal cortex than in the primary visual cortex [44], and can be as long as 1 s in striatal medium spiny neurons [45]. These findings suggest that the prefrontal cortex and the striatum are possible loci of the information decoding.

In psychologic modeling of decision timing, it is hypothesized that the response for a particular decision is generated when the integration of the relevant stimuli reaches a predefined threshold. Response times show trial-by-trial variability even for a constant environment, suggesting that the origin of this variability is internal. Psychologic and neurophysiologic experimental evidence suggests that the variability originates from trial-by-trial fluctuations of the growth rate, but not the threshold, of temporal integration [2],[4],[12],[16],[17]. A random background of excitatory and inhibitory synaptic inputs is unlikely to be the source of these fluctuations, since the effect of such noise is negligible when sufficiently many synaptic inputs are involved in the background noise. Our model predicts that a change in the correlations between input spike trains produces a concomitant change in the growth rate of the temporal integration (Figure 1D), and hence in the response time. Indeed, spiking of multiple cortical neurons is known to be significantly correlated [37]–[40]. On that hypothesis, we conducted numerical simulations to see how the coefficients of variance (i.e., the ratio of the variance to the average) of actual response time depend on the target time. Weber's law in psychology tells that this ratio should remain constant as the target time is varied in the range of second. Our model could replicate this law (Text S1).

How advantageous is it for the brain to perform perfect temporal integration? In the perception of interval timing, perfect integration of constant or regular stimuli over time allows the subject to measure time intervals accurately [54]–[56]. In addition, perfect temporal integration modified with single-trial or trial-by-trial fluctuations of the growth rate can account for the statistical properties of reaction time [2],[12],[16],[17]. In the oculomotor system, perfect integration of eye velocity enables the brain to compute accurate eye positions [57],[58]. The non-leaky property of perfect integration may allow the subject to accumulate and maintain useful information about natural scenes [14],[15]. To our knowledge, the present study is the first to show a neural mechanism of perfect temporal integration.

## Materials and Methods

### Model network

In Equation 1, the leak current is given as 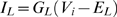, where 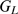 and 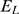 are the conductance and reversal potential, respectively, The neuron fires when the membrane potential 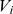 reaches firing threshold 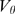; then 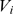 is reset on 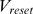 and evolves again according to Equation 1.

The afterdepolarizing current is represented as 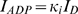 with constant current 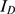. The spike-triggered activation of the depolarizing current and the associated neuronal state transition are modeled as follows. All neurons are initially in the resting state, during which 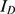 is switched ‘off’ (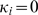 for any 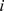). When neuron 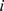 fires, 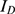 is switched ‘on’ (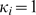) and the neuron is set to the active state. In reality, the afterdepolarizing current may be generated in cortical neurons by activation of Ca^2+^-dependent cation current [33],[34]. We employed the above simplified description to perform an analytic study of the stochastic network dynamics.

The neurons receive 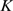 excitatory and 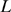 inhibitory external inputs, and are randomly connected by excitatory synapses of an equal maximum conductance, 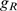. The connectivity of recurrent synapses was set as 20%. In addition, the neuron receives stochastic external synaptic inputs. The recurrent excitatory current and the external inputs are given as
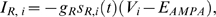
(5)

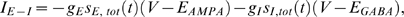
(6)where 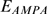 and 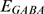 are the reversal potentials of AMPA and GABA_A_ receptor/channel-mediated currents, respectively, and 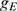 and 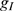 are the maximum conductances of excitatory and inhibitory synapses, respectively. Synaptic activation variables are 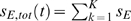, 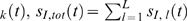 and 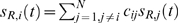, where 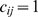 if neuron 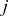 projects to neuron 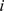. Otherwise, 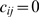. Each gate variable 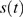 obeys
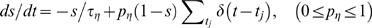
(7)for all types of synapse, where 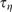 and 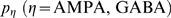 are the decay constant and the release probability of the synapse, respectively, and 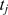 represents the times at which presynaptic neuron 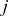 fires. The excitatory and inhibitory external inputs are described as Poisson processes of rates 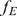 and 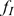, respectively.

### Partially correlated synaptic inputs and Gaussian white-noise approximation

The spike trains arriving at excitatory and inhibitory synapses in Equation 6 are partially correlated, that is, presynaptic spikes are synchronized at 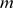 excitatory and 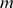 inhibitory synapses with probability 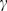. This implies that the spike coincidences occur on average 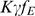 or 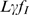 times in 1 second at 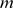 randomly-chosen excitatory or inhibitory synapses, respectively. The Gaussian white-noise approximation of Equation 6 gives a clear insight into the role of partially correlated synaptic inputs [37],[59] in perfect temporal integration by this network model.

Defining the total conductance and the effective reversal potential of synapses as 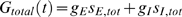 and 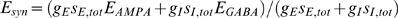, respectively, we can express Equation 6 as 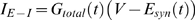, which can be further decomposed into a constant part and a fluctuation component, as shown in Equation 2. The time averages of the total conductance and the effective reversal potential are given as
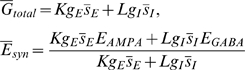
(8a)

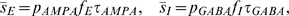
(8b)where Equation 8b was derived from Equation 7 on the assumption that 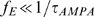 and 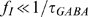.

We can show that 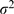 of partially correlated synaptic inputs is given as the following linear function of 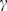:
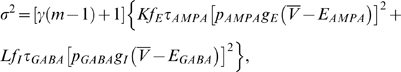
(9)where 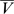 is the time average of the membrane potential. Since 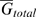 and 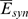 are given as Equations 8a and 8b, respectively, and they are independent of 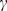, partially correlated synaptic inputs selectively modulate 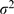 by changing 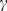. For comparison, if the random presynaptic spike trains processed at individual synapses are mutually uncorrelated, the fluctuation component has mean 0, and the variance is given as
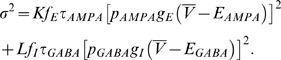
(10)


### The Fokker-Planck approach to the temporal integration process

In general, the fluctuation component 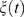 comprises temporally correlated noise (i.e., colored noise). In some of the present analyses, however, we regarded 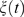 as Gaussian white-noise with mean of 0 and variance of 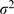, and employed the following Fokker-Planck equation [60] for the probability distribution of the membrane potential, when a neuron is innervated by the external inputs and the recurrent inputs from the surrounding neurons in the active state:
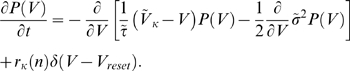
(11)Here, 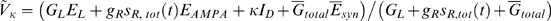, 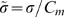, 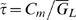, 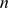 is the instantaneous number of neurons in the active state, and 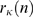 is the inverse of the mean first-passage time that 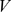 takes to travel from 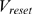 to 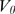 in the resting (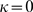) or the active (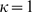) state. Therefore, 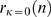 is the average rate of transitions from the resting to the active-state in each neuron, and 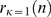 is the mean firing rate of an active-state neuron. The boundary condition is 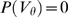.

When the number of active-state neurons is 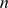 at time 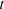, we may replace 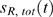 in 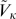 with the average value, 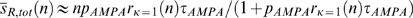. This approximation gives the equilibrium solution to Equation 11 as follows:
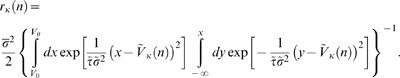
(12)We can recursively solve 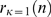 and 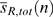 for 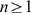 by replacing 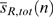 with 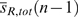 in the r.h.s. of Equation 12 and by using the boundary condition, 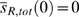. Then, 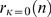 is calculated from 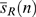 using Equation 12.

### Parameter values

Unless otherwise stated, we use the following parameter values in our simulations: 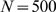; *C_m_* = 0.5 nF; *G_L_* = 20 nS; *I_D_* = 0.12 nA; *τ_AMPA_* = 2 ms; *τ_NMDA_* = 200 ms; *τ_GABA_* = 5 ms; 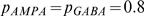; *E_L_* = −70 mV; *E_AMPA_* = 0 mV; *E_GABA_* = −80 mV; *V_θ_* = −52 mV; *V_reset_* = −62 mV in the resting state and *V_reset_* = −54 mV in the active state; *p_AMPA_g_E_* = *p_GABA_g_I_* = 3 nS; *Kf_E_* = 1130 Hz; 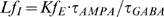; 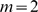.

## Supporting Information

Text S1 This document includes three figures showing temporal integration in a model with realistic intracellular calcium dynamics (Figure S1), possible learning procedure for fine parameter tuning (Figure S2), a numerical proof of Weber's law (Figure S3) and the mathematical details of the model with realistic calcium dynamics.
(0.14 MB PDF)
