# Supplementary material for: Recurrent Network Models for Perfect Temporal Integration of Fluctuating Correlated Inputs
Source: PLoS Comput Biol. 2009 Jun 5;5(6):e1000404. doi: 10.1371/journal.pcbi.1000404 (PMC2685482; doi:10.1371/journal.pcbi.1000404)
Supplement: Text S1 — This document includes three figures showing temporal integration in a model with realistic intracellular calcium dynamics (Figure S1), possible learning procedure for fine parameter tuning (Figure S2), a numerical proof of Weber's law (Figure S3) and the mathematical details of the model with realistic calcium dynamics. (0.14 MB PDF) [file pcbi.1000404.s001.pdf]

Supplementary information

(Okamoto and Fukai)

This document includes three figures about possible learning procedure for fine parameter tuning, numerical proof of Weber's law and the temporal integration performance of our network model with realistic calcium dynamics. The mathematical details of this network model are also described.

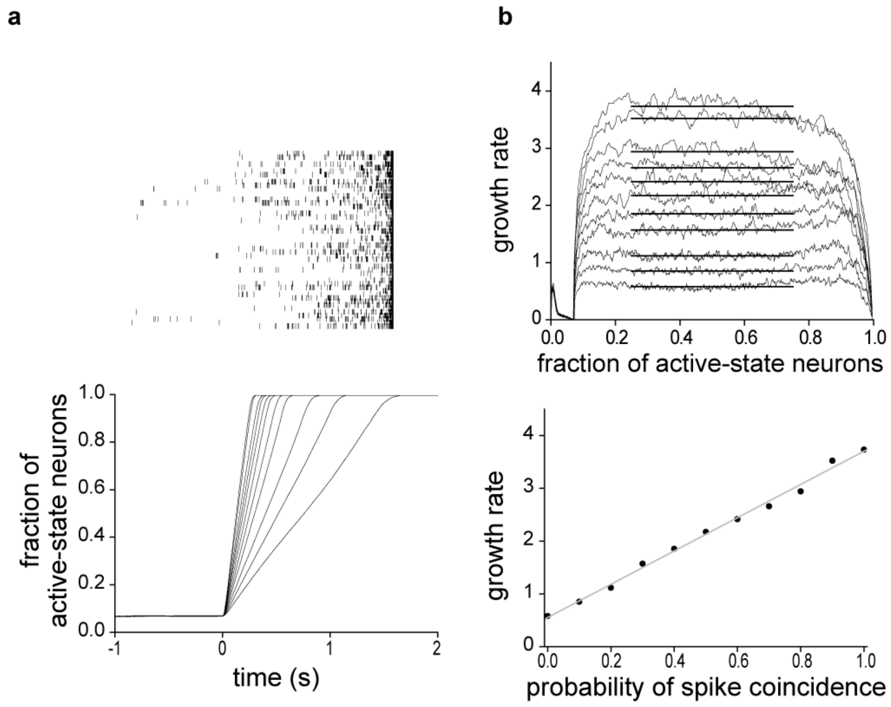

Figure S1: Temporal integration in a model with realistic intracellular calcium dynamics

Calcium dynamics was included into this neuron model to achieve a bistable state in a realistic manner. The details of the network model are shown in the final page of Supplementary Information. *a*, Spike raster of 50 neurons in a single trial (*upper*) and the time evolution of the fraction of active-state neurons (*lower*) are shown. The latter was plotted for several values of the probability of spike coincidence, with each averaged across 100 trials The rates of excitatory and inhibitory synaptic inputs, which has no spike coincidence at $t < 0$, were elevated in a stepwise manner at $t = 0$. Thus, the synaptic inputs exhibited a constant probability of spike coincidences. Note that the spike raster replicated background neuronal firing at $t < 0$ (upper), for which the fraction of active-state neurons remains at a small value (lower) *b*, The growth rate was plotted as a function of the fraction of active-state neurons (*upper*) or the probability of spike coincidence (*lower*). In the latter, the plots were fitted by the least square method (*grey line*).

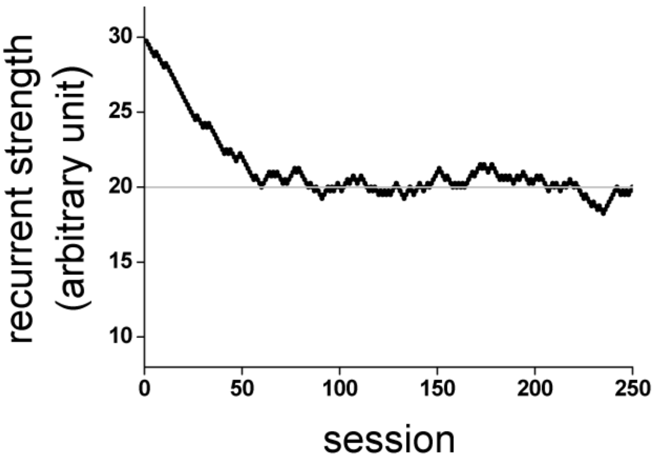

Figure S2: Tuning the strength of recurrent connection in temporal bisection task

Temporal bisection is a psychophysical task widely used to elaborate the percept of interval timing by humans and animals (Church and Deluty 1977; Warden 1991; Allan and Gibbon 1999). In the typical temporal bisection task, two reference intervals, one short (S) and one long (L), are familiarized with by the subject at the beginning of each block of trials. Then, in each test trial, a temporal interval T ($S \le T \le T$) is presented, and the subject is required to judge which interval, S or L, T is closer to. Since humans and animals can solve this task, they are considered to be capable of recognizing the arithmetic mean of two intervals. Noting this fact, we demonstrate how temporal bisection may be used for training the present network, namely, for adjusting the strength of recurrent connection, g_R, in the following simplified version of the bisection task. We trained the network to reproduce the half of the presented interval. Each block of trials consists of a reference trial and a test trial. In the reference trial, a target interval, T_R, was randomly chosen from [0.4 s, 0.8 s]. At $t = 0$, the network started to integrate synaptic inputs of constant intensity with the initial number of active-state neurons being zero. At $t = T_R$, the integration was terminated and the number of active-state neurons, say n_R, was registered. We assumed that the neural system engaging in temporal bisection is pre-programmed to calculate the half of n_R. In the test trial, the network again integrates the same synaptic inputs, and the time point at which the number of active-state neurons reached $n_R/2$ was employed as the subjective bisection time point $t_{\text{subjective}}$. Note that if the temporal integration performed by the network is perfect, $t_{\text{subjective}} = T_R/2$. After the test trial, the strength of recurrent connection was modified as $g_R \to g_R + \varepsilon\Delta_g$, where $\varepsilon = +1$ if $t_{\text{subjective}} > T_R/2$ or $\varepsilon = -1$ if $t_{\text{subjective}} < T_R/2$. A similar block is repeated sufficiently many times until g_R converges to a value at which the network accurately perform temporal bisection (*grey line*). In the present simulations, $\Delta_g = 0.25$ (arbitrary unit) and the initial value (before learning) was $g_R = 30$ (arbitrary unit).

References

Allan LG., Gibbon J (1999) Human bisection at the geometric mean. *Learning & Motivation* 22: 39-58.

Church RM, Deluty MZ (1977) Bisection of temporal intervals. *Journal of Experimental Psychology: Animal Behavior Processes* 3: 216-228.

Wearden JH (1991) Human performance on an analogue of an interval bisection task. *Quarterly Journal of Experimental Psychology* 43B: 59-81.

(a) (b)

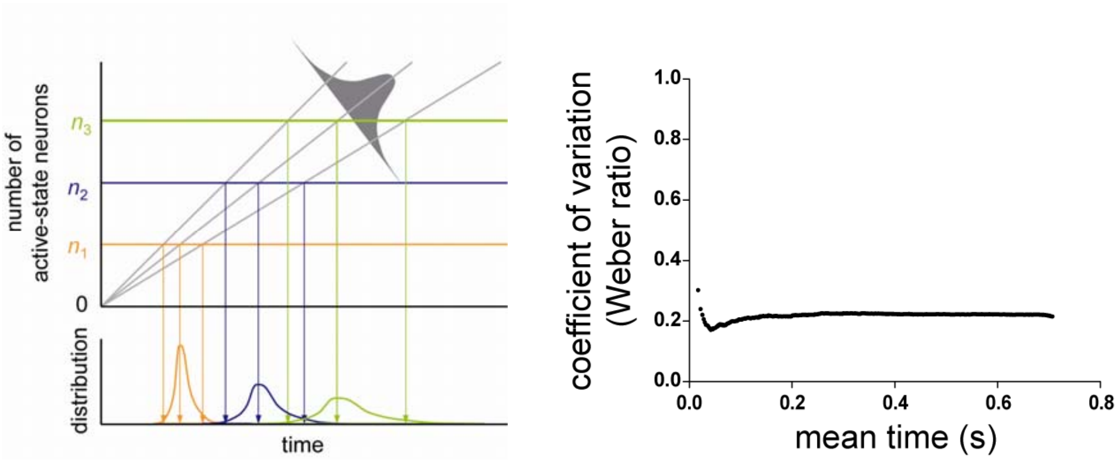

Figure S3: **Weber's law obeyed by the network model**

(a) Schematic illustration of Weber's law. In reality, the rate of temporal integration varies from trial to trial, so does the judgement of a time interval. In the simulations shown here, the probability of spike coincidences γ in each trial was variable and was determined according to a Gaussian distribution,

$$P(\gamma) = \frac{1}{\sqrt{2\pi\sigma_\gamma^2}} \exp\left(-\frac{(\gamma - \gamma_0)^2}{2\sigma_\gamma^2} \right) ,$$

with $\gamma_0 = 0.3$ and $\sigma_\gamma = 0.1$, and the average μ and variance σ of the time needed to activate a given number of neurons were measured. The simulations were repeated for different target numbers of neurons. (b) The coefficient of variation σ/μ obtained by the simulations is shown. The constancy of the plot implies Weber's law.

Neuron model with realistic calcium dynamics

ADP current was modelled as $I_{ADP} = G_{ADP} P_{ADP} \left(V - E_{ADP}\right)$, where P_{ADP} is the activation probability of the current:

$$\text{close} \underset{k_{off}}{\overset{k_{on}\left([\text{Ca}^{2+}]\right)}{\rightleftarrows}} \text{open}$$

$$\frac{dP_{ADP}}{dt} = k_{on}\left([\text{Ca}^{2+}]\right)\left(1 - P_{ADP}\right) - k_{off} P_{ADP} \text{ with } k_{on}\left([\text{Ca}^{2+}]\right) = \frac{[\text{Ca}^{2+}]^4}{[\text{Ca}^{2+}]^4 + K_{Ca}{}^4}.$$

Intracellular calcium density $[\text{Ca}^{2+}]$ obeys

$$\frac{d\left[\text{Ca}^{2+}\right]}{dt} = -\frac{1}{\tau_{Ca}}\left[\text{Ca}^{2+}\right] + \Delta_{Ca}\sum_{t_s}\delta\left(t - t_s\right),$$

where the second term describes the spike-triggered calcium entry to cell body. Parameter values were set as $\tau_{Ca} = 0.07$ sec, $\Delta_{Ca} = 0.5$ (scaled unit); $G_{ADP} = 6\times10^{-9}$ S, $E_{ADP} = -0.035$ V, $k_{on} = 0.001$ sec, $k_{off} = 0.3$ sec, $K_{Ca} = 1$ (scaled unit). Since $0 \le P_{ADP} \le 1$ and P_{ADP} is close to unity in an actively firing neuron, the number of active-state neurons was defined as $\sum_{n=1}^{N} P_{ADP,\,n}$.
